# Delayed Endobronchial Metastasis of Colorectal Adenocarcinoma Five Years After Resection: A Case Report

**DOI:** 10.7759/cureus.108561

**Published:** 2026-05-09

**Authors:** Wesley Chow, Sevag Hamamah, Garrett Teskey, Luke Shenton, Julian Lichter

**Affiliations:** 1 Internal Medicine, Scripps Mercy Hospital, San Diego, USA; 2 Critical Care and Pulmonology, Scripps Mercy Hospital, San Diego, USA

**Keywords:** bronchoscopic mass resection, colorectal carcinoma, colorectal carcinoma metastasis, colorectal carcinoma surveillance, delayed recurrence, endobronchial metastasis, partial airway obstruction, unique adrenal metastasis

## Abstract

Endobronchial metastasis (EBM) is a rare manifestation of extrapulmonary malignancies and poses a diagnostic challenge because it can closely mimic primary bronchogenic carcinoma. Although colorectal carcinoma (CRC) is among the most common malignancies worldwide, endobronchial involvement is distinctly rare and represents an atypical pattern of metastatic dissemination. We present the case of an 83-year-old woman with a history of resected KRAS-mutant sigmoid adenocarcinoma who developed symptomatic near-complete endobronchial obstruction more than five years after complete resection by colectomy. Imaging demonstrated presumed widespread multiorgan metastatic disease, including a large left upper lobe mass, mediastinal lymphadenopathy, hepatic and adrenal lesions, vertebral lytic lesions, and bilateral pulmonary nodules. Bronchoscopy revealed an exophytic mass in the left mainstem bronchus, causing near-complete luminal obstruction. Multimodal endoscopic debulking using a cautery snare, argon plasma coagulation (APC), and laser ablation successfully restored airway patency. Histopathology confirmed moderately differentiated metastatic colonic adenocarcinoma with CK20 and CDX2 positivity. This case highlights that CRC can recur with aggressive dissemination to rare metastatic sites even after complete surgical resection. Furthermore, loss to follow-up in high-risk patients may permit undetected disease progression, supporting a more individualized approach to long-term surveillance. Imaging alone may fail to detect endobronchial disease, making early bronchoscopy essential for timely diagnosis and management.

## Introduction

Endobronchial metastasis (EBM) refers to malignant infiltration of the bronchial lumen by extrapulmonary cancers and accounts for 1%-4% of all endobronchial tumors [1,2]. Over the past several decades, EBM has been increasingly recognized due to advances in bronchoscopic techniques and cross-sectional imaging [3]. Clinically, EBM can closely resemble primary bronchogenic carcinoma, presenting with cough, dyspnea, hemoptysis, or post-obstructive pneumonia, thereby posing a significant diagnostic challenge that requires histopathologic confirmation [4,5].

While primary bronchogenic carcinoma remains the most common cause of endobronchial tumors, metastatic involvement of the bronchial lumen from extrapulmonary malignancies has been well documented across a range of primary cancers, most commonly breast cancer, followed by colorectal carcinoma (CRC), renal cell carcinoma, prostate cancer, and melanoma [2-4]. The timing of EBM relative to the primary diagnosis is variable. In some series, endobronchial lesions are identified synchronously with the primary tumor, whereas in others, they appear years to decades after initial resection, complicating long-term surveillance strategies [2,3,6].

CRC is the third most common malignancy worldwide and remains a leading cause of cancer-related mortality [7]. The most frequent metastatic sites of CRC are the liver, lungs, and peritoneum, reflecting patterns of hematogenous and lymphatic dissemination. Pulmonary parenchymal metastases occur in approximately 10%-15% of patients with CRC; however, endobronchial involvement is distinctly rare and has been reported in fewer than 2% of metastatic CRC cases [2]. The pathophysiology of EBM in CRC is not fully established but is thought to involve hematogenous seeding of the bronchial mucosa, submucosal lymphatic spread, or direct extension from peribronchial lymph node metastases [8].

Here, we present a rare case of delayed EBM from CRC manifesting as near-complete airway obstruction more than five years after complete resection, with concurrent multi-organ metastatic involvement managed through multimodal bronchoscopic intervention.

## Case presentation

An 83-year-old woman presented with one month of persistent nonproductive cough, dyspnea, hoarseness, and unintentional 10-pound weight loss. She denied fever, hemoptysis, chest pain, or other systemic symptoms. On examination, she was breathing comfortably on room air, and lung auscultation was unremarkable. Prior to presentation, she had completed a course of empiric antibiotics and corticosteroids for presumed pneumonia without symptomatic improvement. 

Her medical history included sigmoid adenocarcinoma diagnosed five years earlier in Mexico, for which she underwent surgical resection, followed by six months of capecitabine therapy. Pathology confirmed colonic adenocarcinoma (pT3N0M0, Stage IIA) with 0 of 18 lymph nodes involved, no lymphovascular or perineural invasion, negative surgical margins, and a KRAS mutation. One-year follow-up colonoscopy and carcinoembryonic antigen (CEA) testing were unremarkable, after which she was lost to follow-up. Additional history included former tobacco use, breast cancer treated with lumpectomy 24 years earlier, and unspecified skin cancer treated with Mohs surgery three years prior. Family history was noncontributory.

**Figure 1:**
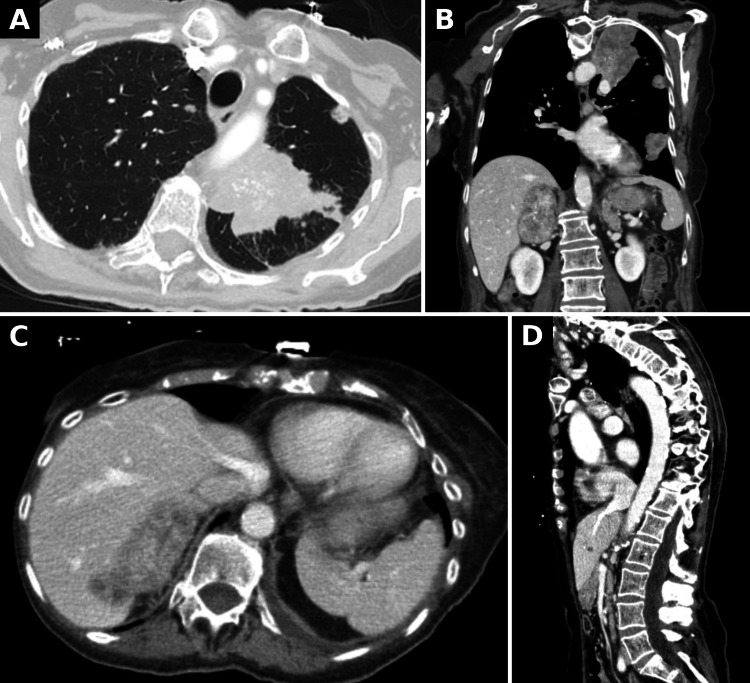
Computed tomography (CT) of the chest, abdomen, and pelvis demonstrating a left upper lobe pulmonary mass (A), bilateral heterogeneous adrenal masses (B), hepatic lesion (C), and thoracic spine lytic lesions (D)

**Figure 2:**
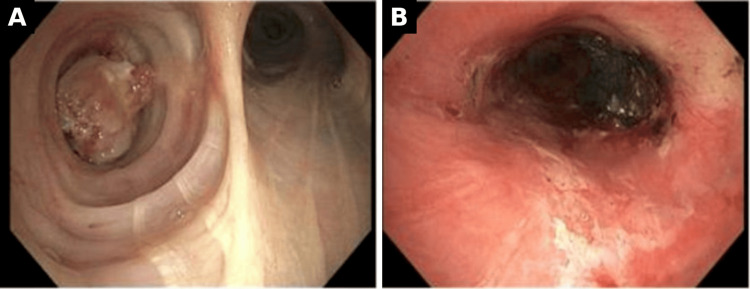
Bronchoscopic findings demonstrating an exophytic mass obstructing the proximal left mainstem bronchus before intervention (A) and restoration of airway patency following tumor excision (B)

**Figure 3:**
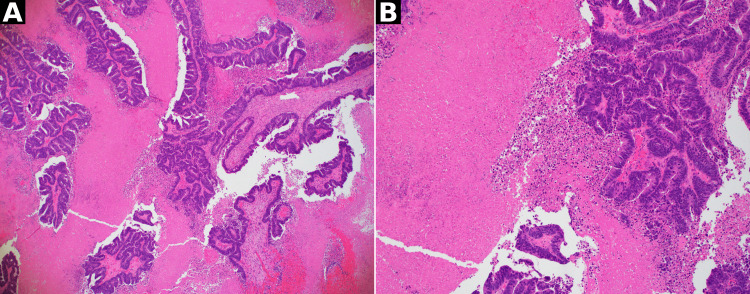
Histopathologic findings consistent with metastatic colonic adenocarcinoma: low-power view demonstrating adenocarcinoma (A) and high-power view demonstrating characteristic “dirty necrosis” typical of colonic adenocarcinoma (B)

Routine laboratory studies, including complete blood count and comprehensive metabolic panel, were within normal limits. Chest radiography revealed multiple bilateral pulmonary masses. Subsequent computed tomography (CT) imaging demonstrated a large 7.0 × 5.5 cm left upper lobe pulmonary mass with multiple bilateral pulmonary nodules, bulky mediastinal lymphadenopathy, vertebral lytic lesions, a 2.5 cm hypoattenuating hepatic lesion, a 7.0 × 4.5 cm right adrenal mass, and a 3 cm left adrenal mass (Figures 1A-1D). CEA was markedly elevated at 1,282 ng/mL. Given the imaging findings and the inability to exclude a primary lung malignancy, bronchoscopy was performed and identified near-complete obstruction of the left mainstem bronchus by an exophytic mass (Figure 2A). Tumor debulking using a cautery snare, argon plasma coagulation (APC), and laser ablation successfully restored airway patency (Figure 2B). Biopsy revealed moderately differentiated adenocarcinoma with intestinal features that was CK20- and CDX2-positive and CK7- and TTF-1-negative, consistent with colorectal origin (Figure 3). Attempts at PCR-based molecular analysis were limited by insufficient tissue from the biopsy specimens. Her clinical status remained stable, and she was discharged with plans for outpatient oncology follow-up to pursue palliative management aligned with her goals of quality-focused care in the setting of advanced disease.

## Discussion

This case is notable for the extent of presumed systemic dissemination at presentation, with imaging suggesting concurrent involvement of the liver, spine, and bronchial lumen. While hepatic and pulmonary parenchymal metastases are common in advanced CRC, simultaneous endobronchial and vertebral involvement is uncommon and reflects aggressive systemic dissemination [9]. Importantly, histologic confirmation was obtained only for the endobronchial lesion, while the remaining sites of involvement were characterized radiographically. Despite this limitation, the constellation of findings in a patient with known KRAS-mutant CRC and markedly elevated CEA provides a compelling clinical picture of widespread metastatic disease, further supported by the endobronchial biopsy findings.

Radiographic evaluation of suspected endobronchial disease is inherently limited, as cross-sectional imaging may not fully characterize lesions within the bronchial lumen, particularly when obstruction is partial or obscured by adjacent parenchymal changes [10]. In this patient, CT imaging identified a large left upper lobe mass and extensive mediastinal lymphadenopathy, but could not definitively distinguish EBM from a primary lung malignancy. This distinction carries significant therapeutic implications, as management of metastatic CRC differs substantially from that of primary bronchogenic carcinoma. Bronchoscopy was therefore essential, providing direct visualization for precise assessment of lesion location, extent, and degree of luminal compromise. Visual features, including tumor vascularity, friability, and exophytic growth pattern, informed decisions regarding resection technique and bleeding risk, reinforcing that bronchoscopy in this setting serves not only as a diagnostic tool but also as a critical component of procedural planning and risk stratification.

Endoscopic intervention was central to this patient’s acute management. Snare excision, APC, and neodymium-doped yttrium aluminum garnet (Nd:YAG) laser ablation are commonly used techniques for endobronchial debulking [11]. Thermal modalities carry inherent risks, including airway wall injury, perforation, and ignition in high-oxygen environments and therefore must be applied cautiously to avoid collateral damage to adjacent bronchial structures [12,13]. In this patient, all three modalities were applied sequentially with close attention to these risks, achieving immediate luminal patency and restoration of airflow without procedural complications. This case illustrates that even near-complete endobronchial obstruction can be effectively managed bronchoscopically, providing meaningful palliation and improved quality of life even in the setting of advanced systemic disease when surgical resection is not feasible.

Molecular profiling of CRC has enhanced understanding of the prognostic and predictive implications of specific oncogenic alterations. KRAS mutations, present in approximately 35%-45% of CRCs, are associated with a more aggressive biological phenotype [14]. This case raises the question of whether KRAS-mutant tumors may carry a greater propensity for delayed dissemination to unusual anatomical sites, including endobronchial spread. However, this risk is not attributable to KRAS alone, as the BRAF V600E mutation independently confers poorer outcomes and higher recurrence rates [15]. Alternatively, microsatellite instability (MSI) status, generally considered protective in early-stage disease, has been associated with distinct recurrence patterns and paradoxically worse post-recurrence survival, underscoring the complex and context-dependent prognostic landscape of these molecular markers [16]. Collectively, these molecular risk factors may help explain the significant metastatic burden observed in this case. However, repeat molecular analysis at the time of recurrence was limited by insufficient biopsy tissue, precluding direct comparison of mutational profiles between the primary and metastatic lesions to determine whether acquisition of more aggressive molecular phenotypes contributed to relapse.

The majority of CRC recurrences occur within the first three years following resection, prompting guideline-recommended surveillance strategies that emphasize intensive monitoring during this high-risk period before transitioning to less frequent intervals through the fifth postoperative year [17]. However, emerging data suggest that a clinically meaningful proportion of recurrences occur beyond this period, particularly among patients with high-risk molecular features or interrupted surveillance [18]. This patient received only one year of post-resection surveillance before being lost to follow-up, which falls well short of the three-to-five-year monitoring period recommended by current CRC guidelines. Without continued surveillance, the exact timing of recurrence remains unknown. Although she presented symptomatically five years after resection, subclinical disease may have developed considerably earlier, potentially within the three-to-five-year interval during which recurrence is most common in CRC. This underscores the importance of uninterrupted surveillance, as earlier detection during an asymptomatic phase may have altered the clinical trajectory.

## Conclusions

This case underscores that CRC can recur at rare anatomical sites with aggressive systemic dissemination, warranting a high index of clinical suspicion in patients with a history of CRC who present with new respiratory symptoms. When cross-sectional imaging is insufficient to characterize endobronchial disease or exclude a primary lung malignancy, early bronchoscopy is essential as both a diagnostic and therapeutic modality capable of restoring airway patency even in advanced disease. Additionally, this case highlights how loss to follow-up after only one year of post-resection surveillance may contribute to delayed recognition of recurrence, underscoring the need for individualized and potentially extended surveillance strategies in patients with high-risk molecular profiles or limited prior access to structured oncologic care.
